# Draft Genome Sequences of Three Listeria monocytogenes Strains Isolated from Chicken Carcasses in South Korea

**DOI:** 10.1128/mra.01249-22

**Published:** 2023-02-22

**Authors:** Sangmi Lee, Sangryeol Ryu

**Affiliations:** a Department of Food and Nutrition, Chungbuk National University, Cheongju, Chungbuk, South Korea; b Department of Food and Animal Biotechnology, Seoul National University, Seoul, South Korea; University of Rochester School of Medicine and Dentistry

## Abstract

While Listeria monocytogenes is a foodborne pathogen of global concern, little is known regarding L. monocytogenes populations in Asia. We report the draft genome sequences of three L. monocytogenes strains from chickens in South Korea, which could facilitate understanding the genetic diversity of the regional L. monocytogenes population.

## ANNOUNCEMENT

Listeria monocytogenes causes listeriosis in humans, which exhibits high hospitalization and mortality rates along with severe symptoms (1–4). Listeriosis is caused by the consumption of contaminated foods, and L. monocytogenes has been found in various environments (5–8). To date, major listeriosis outbreaks have been reported in Europe and North America, resulting in extensive characterization of strains from those regions, while strains isolated in Asia remain poorly understood (8–12). Here, we report the whole-genome sequences of three L. monocytogenes strains (SNU7, SNU8, and SNU27) that were isolated in Seoul, South Korea (13).

The strains were isolated from chicken carcasses in 2004 as recommended by the U.S. Food and Drug Administration (13) and stored at −80°C. Cells were streaked from cryostocks onto brain heart infusion (BHI) (Becton, Dickinson and Co., Sparks, MD, USA) agar plates and incubated overnight at 37°C. Genomic DNA was extracted from cells collected from the BHI agar plates with the NucleoSpin microbial DNA kit (Macherey-Nagel, Düren, Germany) and was utilized to prepare paired-end libraries with the TruSeq Nano DNA library preparation kit (Illumina, San Diego, CA, USA). The genomes were sequenced with a MiSeq system (2 × 300 bp reads; Illumina) (Table 1), and raw sequencing reads were quality checked with FastQC v0.11.9 (http://www.bioinformatics.babraham.ac.uk/projects/fastqc), which was also employed to examine overrepresented sequences. Using Trimmomatic v0.38 (14), adaptors were trimmed and low-quality reads (with quality scores of <20) were removed. Then, sequences from the PhiX Control v3 library (Illumina) used as the sequencing control were removed with BWA v0.7.17 (15), resulting in 648,634 to 954,041 pairs of reads, which were subsequently *de novo* assembled using SPAdes v3.13.0 (16) (Table 1). To identify multilocus sequence typing (MLST)-based sequence type (ST) and clonal complex (CC) designations, the draft genomes were subjected to *in silico* MLST analysis provided by BIGSdb-Lm, the *Listeria* MLST database maintained by Institut Pasteur (https://bigsdb.pasteur.fr/listeria). This analysis indicated that strains SNU7 and SNU8 belonged to ST9 CC9, while strain SNU27 was classified as ST224 CC224. The contigs for each strain were annotated via the NCBI Prokaryotic Genome Annotation Pipeline (PGAP) v6.3 (17) (Table 1). The genome sequences reported in this announcement will deepen understanding of the genetic diversity of L. monocytogenes populations from overlooked regions such as Asia.

**TABLE 1:** Characteristics of the genome data

Characteristic	Data for strain:		
	SNU7	SNU8	SNU27
No. of read pairs	1,568,942	1,498,460	1,177,830
Total length of reads (Mbp)	843.3	791.2	626.3
Draft genome length (Mbp)	2.96	2.94	2.91
Avg coverage (×)	167.81	139.4	113.23
Avg GC content (%)	37.84	37.81	37.88
No. of contigs	17	9	8
*N*_50_ (bp)	430,382	605,099	511,269
No. of protein-coding sequences	2,932	2,865	2,803
No. of pseudogenes	16	12	20
No. of rRNAs	6	5	7
No. of tRNAs	54	47	54

### Data availability.

The draft genome sequences of SNU7, SNU8, and SNU27 have been deposited in GenBank under the accession numbers JAPHNT000000000, JAPHNU000000000, and JAPHNV000000000, respectively (BioProject accession number PRJNA898764, with BioSample accession numbers SAMN31666075 to SAMN31666077 and SRA accession numbers SRR22356288 to SRR22356286). The versions described in this paper are the first versions.
